# Clinical Utility of Serial Measurements of Antineutrophil Cytoplasmic Antibodies Targeting Proteinase 3 in ANCA-Associated Vasculitis

**DOI:** 10.3389/fimmu.2020.02053

**Published:** 2020-09-03

**Authors:** Gwen E. Thompson, Lynn A. Fussner, Amber M. Hummel, Darrell R. Schroeder, Francisco Silva, Melissa R. Snyder, Carol A. Langford, Peter A. Merkel, Paul A. Monach, Philip Seo, Robert F. Spiera, E. William St. Clair, John H. Stone, Ulrich Specks

**Affiliations:** ^1^Essentia Health, Division of Pulmonary and Critical Care, Fargo, ND, United States; ^2^Mayo Clinic and Mayo Foundation for Research and Education, Rochester, MN, United States; ^3^Division of Pulmonary, Critical Care, and Sleep Medicine, The Ohio State University, Columbus, OH, United States; ^4^Department of Rheumatology, Facultad de Medicina Clínica Alemana Universidad del Desarrollo, Santiago, Chile; ^5^Cleveland Clinic, Division of Rheumatology, Cleveland, OH, United States; ^6^Division of Rheumatology, Department of Medicine, University of Pennsylvania, Philadelphia, PA, United States; ^7^Division of Clinical Epidemiology, Department of Biostatistics, Epidemiology, and Informatics, University of Pennsylvania, Philadelphia, PA, United States; ^8^Division of Rheumatology, Brigham and Women's Hospital, Boston, MA, United States; ^9^Division of Rheumatology, Johns Hopkins University, Baltimore, MD, United States; ^10^Division of Rheumatology, Hospital for Special Surgery, New York, NY, United States; ^11^Division of Rheumatology, Duke University, Durham, NC, United States; ^12^Division of Rheumatology, Massachusetts General Hospital, Boston, MA, United States

**Keywords:** ANCA-associated vasculitis, PR3-ANCA, remission, relapse activity, biomarker

## Abstract

**Background:** The utility of ANCA testing as an indicator of disease activity in ANCA-associated vasculitis (AAV) remains controversial. This study aimed to determine the association of ANCA testing by various methods and subsequent remission and examine the utility of a widely used automated addressable laser-bead immunoassay (ALBIA) to predict disease relapses.

**Methods:** Data from the Rituximab vs. Cyclophosphamide for ANCA-Associated Vasculitis (RAVE) trial were used. ANCA testing was performed by direct ELISA, capture ELISA, and ALBIA. Cox proportional hazards regression models were used to evaluate the association of PR3-ANCA level and subsequent remission or relapse. The ALBIA results are routinely reported as >8 when the value is high. For this study, samples were further titrated. A decrease and increase in PR3-ANCA were defined as a halving or doubling in value, respectively.

**Results:** A decrease in ANCA by ALBIA at 2 months was associated with shorter time to sustained remission (HR 4.52, *p* = 0.035). A decrease in ANCA by direct ELISA at 4 months was associated with decreased time to sustained remission (HR 1.77, *p* = 0.050). There were no other associations between ANCA decreases or negativity and time to remission. An increase in PR3-ANCA by ALBIA was found in 78 of 93 subjects (84%). Eleven (14%) had a PR3-ANCA value which required titration for detection of an increase. An increase of ANCA by ALBIA was associated with severe relapse across various subgroups.

**Conclusions:** A decrease in ANCA by ALBIA at 2 months and by direct ELISA at 4 months may be predictive of subsequent remission. These results should be confirmed in a separate cohort with similarly protocolized sample and clinical data collection. A routinely used automated ALBIA for PR3-ANCA measurement is comparable to direct ELISA in predicting relapse in PR3-AAV. Without titration, 14% of the increases detected by ALBIA would have been missed. Titration is recommended when this assay is used for disease monitoring. The association of an increase in PR3-ANCA with the risk of subsequent relapse remains complex and is affected by disease phenotype and remission induction agent.

## Introduction

The antineutrophil cytoplasmic antibody (ANCA)-associated vasculitides (AAV) are characterized by necrotizing inflammation affecting predominantly small vessels (1). Three conditions comprise AAV: granulomatosis with polyangiitis (GPA), microscopic polyangiitis (MPA), and eosinophilic granulomatosis with polyangiitis (EGPA). GPA and MPA have many clinical similarities and have been studied in the same clinical trials, whereas EGPA has been excluded from these studies (2–6).

Historically, the course of AAV was inevitably fatal. The advent of treatment with immunosuppressive regimens such as cyclophosphamide (CYC) or rituximab (RTX), in combinationwith glucocorticoids (GCS), changed this course to one of a chronic relapsing disease. Remission is achievable with induction therapy in 70–90% of patients but more than half of patients in remission are at risk for relapse, particularly if they have ANCA reacting with proteinase 3 (PR3-ANCA) (7). Morbidity and mortality in AAV not only occurs from the disease process itself but also from complications secondary to immunosuppression. Therefore, balancing the risks of immunosuppression with the need for disease control is imperative, and accurate prediction of relapses is an important contributor to this balance (8–10).

Ever since the discovery of ANCA, the utility of ANCA testing as an indicator of disease activity or prediction tool of relapse has been investigated with conflicting results (11–34). To date, there has not been evidence that decreases in ANCA levels indicate subsequent remission (27). More recent studies demonstrating an association between rising PR3-ANCA levels and risk of relapse showed such associations to be dependent on ANCA-detection methodology, disease phenotype, and treatment regimen (34, 35). The current study aimed to determine the association of ANCA testing by various methods and subsequent remission. As ANCA-test methodologies are evolving, and automated addressable laser-bead immunoassay (ALBIA) ANCA testing platforms are more widely used in high volume laboratories, this study also aimed to examine the utility of an ALBIA for relapse prediction in AAV patients in comparison to methods previously reported.

## Methods

### Patients

Serum samples and clinical data from the Rituximab vs. Cyclophosphamide for ANCA-Associated Vasculitis (RAVE) trial were used (3). All patients had provided consent for the use of both serum samples and clinical data collected during the RAVE trial for subsequent ancillary studies. The RAVE trial was approved by the Institutional Review Boards of each participating center.

Details of the RAVE trial are described elsewhere (3, 36). Briefly, RAVE was a multicenter, randomized, double-blind, double placebo-controlled trial design that included 197 patients with severe, ANCA-positive GPA or MPA. Of the 197 patients, 131 were PR3-ANCA positive and 66 were MPO-ANCA positive. The patients were randomized 1:1 to either RTX with 4 weekly treatments of 375 mg/m^2^ or oral CYC of 2 mg/kg/day for 3–6 months followed by Azathioprine (AZA) to month 18. All patients received GCS consisting of intravenous methylprednisolone followed by a prednisone taper. The follow-up protocol consisted of visits at baseline; then weekly for 4 weeks, then monthly until 6 months, then every 3 months until month 18. Subsequently, patients were seen every 6 months until trial closeout which occurred 18 months after the last patient was enrolled. Additional study visits occurred at the patients' and providers' discretion, usually in the case of disease relapse or serious adverse event (3, 36).

It has been established that patients with PR3-ANCA are at increased risk of relapse compared to MPO-ANCA positive patients (7, 33, 36–41). The combination of a lower number of MPO-ANCA patients enrolled in the RAVE trial with less frequent relapses in this population resulted in a small sample of relapsing MPO-ANCA patients (3, 36). For this reason, PR3-ANCA positive patients were the population of interest for the current study.

### Disease Activity and Phenotype

Assessment of disease activity was completed at each study visit using the BVAS/WG instrument with active disease defined as a score of ≥1 and a score of 0 reflecting remission (42). Complete remission was defined as a BVAS/WG of 0 with a prednisone dose of 0 mg. Sustained remission was defined as a BVAS/WG of 0 with a prednisone dose of 0 mg for 6 months. A patient was considered to relapse if there was an increase of BVAS/WG of ≥1 after achievement of complete remission. A severe relapse was defined as an increase in BVAS/WG of >3, new major item on BVAS/WG, or if induction therapy was reinitiated per clinician discretion (43).

Organ manifestations were recorded at enrollment and at each study visit with the BVAS/WG. The disease phenotype used for these analyses are based on that present at the time of enrollment. Patients were categorized into 1 or more of 5 groups: granulomatous disease only, any granulomatous disease, any capillaritis, renal involvement, and alveolar hemorrhage. These partially overlapping categories are described in detail elsewhere (35).

### ANCA Testing

ANCA testing was performed using standardized direct enzyme-linked immunosorbent assays (ELISAs) for PR3-ANCA and MPO-ANCA (supplied by Euroimmun) on all baseline serum samples (28). If found to be ANCA positive, serial samples were tested using multiple methods including direct ELISA, capture ELISA (44), and an automated addressable laser-bead immunoassay (ALBIA) (BioPlex 2200, Biorad) (45). Serum samples for each patient were run together at a single laboratory from the second thaw cycle of each sample for each visit.

A PR3-ANCA test result obtained by ALBIA is considered equivocal if a value of 0.4–0.9. In this study, if a value was within the equivocal range the presence of a cANCA pattern was confirmed by immunofluorescence. This method increases the sensitivity of the ALBIA assay without compromising specificity. Results of the ALBIA assay are routinely reported to a value of 8 units, after which it is reported as >8 units. For this study, samples were additionally titrated 1:1, 1:4, 1:10, and 1:100 with the highest value recorded. If a value was not titrated, then the first titrated value was used. An increased PR3-ANCA level was defined as doubling in value compared to the lowest visit in the last 6 months. A decreased ANCA level was defined as halving of value compared to the highest visit in the last 6 months. All changes were outside the intra-assay and inter-assay coefficients of variation.

### Statistical Analyses

Statistical analyses were completed using SAS, version 9.3 (SAS Institute). Descriptive data are reported as mean (SD), median and percentages.

#### PR3-ANCA Levels and Remission

This analysis was based on all patients who achieved complete remission following remission induction therapy (*n* = 108) whether achieved on originally assigned treatment or after cross-over. Cox proportional hazards models were used to assess whether ANCA level decreases were associated with subsequent remission. Analysis was completed looking at ANCA decrease for the event of interest of complete remission or sustained remission. To examine whether a decrease in ANCA was associated with remission, the times of interest were 2 and 4 months after enrollment in the study (time 0). Patients were classified according to their ANCA as “decrease” or “no decrease” Patients classified as “no decrease” included all patients that did not meet criteria for a decrease as defined as halving of ANCA value including those who had an increase in ANCA, had stable ANCA levels and had a decrease in ANCA that did not meet criteria. Time to complete remission and sustained remission were then estimated by using the Kaplan-Meier method.

All analyses were performed for the entire cohort and for patient subsets defined according to disease phenotype and treatment groups. *P* < 0.05 were considered statistically significant.

#### PR3-ANCA Levels and Relapse

This analysis was based on patients who achieved complete remission with the originally assigned remission induction treatment only (*n* = 93). Cox proportional hazards models were also used to assess whether PR3-ANCA level increases were associated with subsequent relapse. This analysis was completed looking at a rise in PR3-ANCA for the event of interest of “any” relapse and “severe” relapse. Hazard ratios with corresponding 95% confidence intervals were used to quantify the increase in risk of relapse within 12 months after a PR3-ANCA increase. In order to determine the accuracy of the model to discriminate patients at increased risk of relapse, c-indices were calculated as was completed in a previous study (35). A c-index of 0.5 indicates no discrimination, and a value of 0.7–0.8 indicates adequate discrimination (46).

Increase in PR3-ANCA was examined using a binary time-varying covariate with the variable having a value of “0” from the time of complete remission to the date that an increase in PR3-ANCA occurred and a value of “1” following an increase in PR3-ANCA. With this method, if an increase occurred at the time of a relapse it would not be detected. Kaplan-Meier analysis that included patients with an increase in PR3-ANCA was completed with time 0 defined as the time of increase.

## Results

### Correlation of Direct ELISA and Automated ALBIA Results

**Figure 1:**
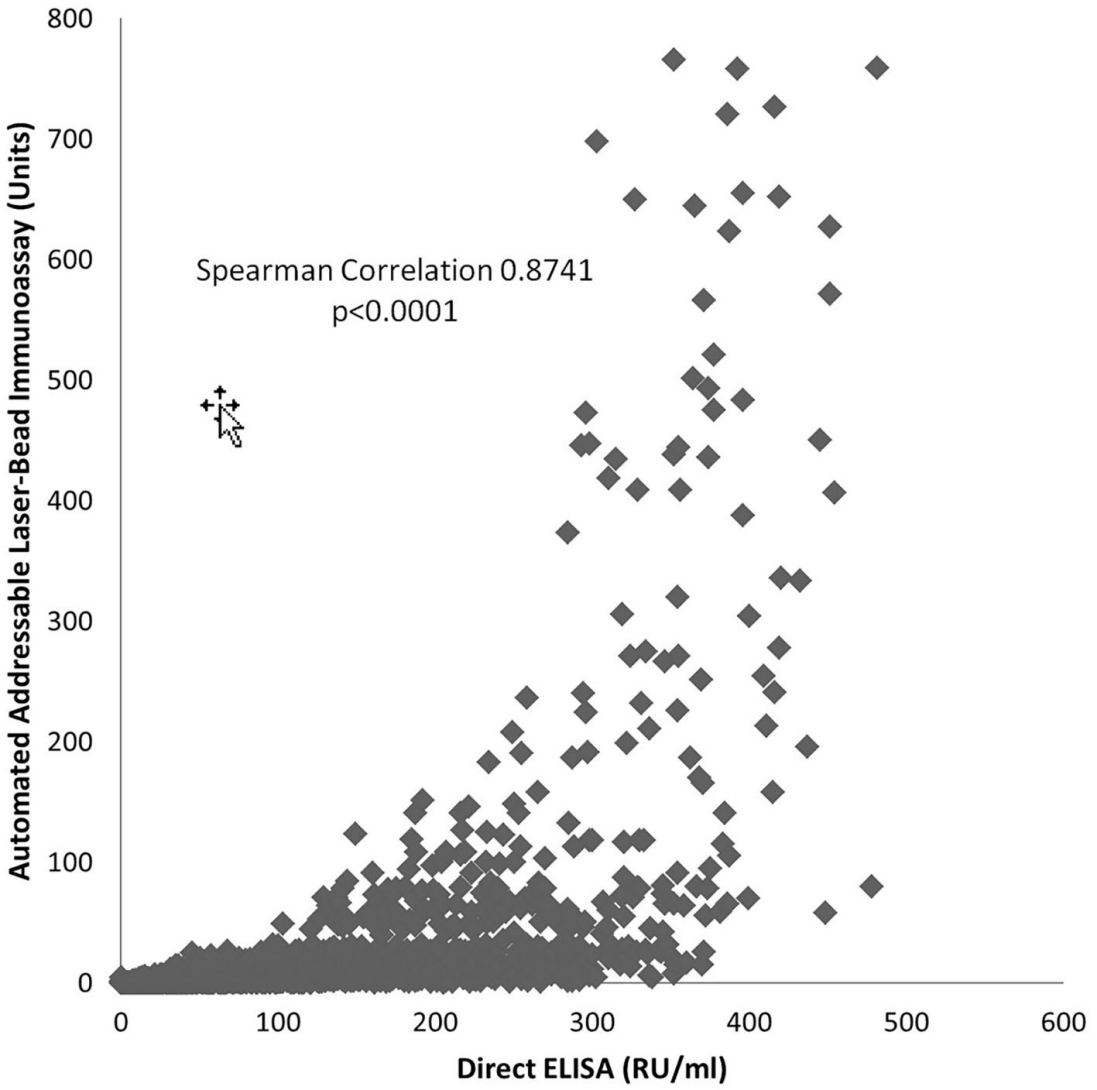
Relationship of PR3-ANCA Measurement by Automated Addressable Laser-Bead Immunoassay and Direct ELISA.

Results obtained with the direct ELISA and the automated ALBIA are positively correlated (Spearman 0.8741, *p* < 0.0001); therefore, with an increase in one an increase in the other is expected, and the opposite is true (Figure 1). As the tests do not have a correlation coefficient of 1.0, a change by a certain increment in one will not result in that same degree of change in the other test.

### PR3-ANCA Titer Decreases and Subsequent Remission

The baseline characteristics of the 131 PR3-ANCA positive participants of the RAVE trial have been described elsewhere (47). The median time from enrollment to remission, complete remission, and sustained remission were 2, 6, and 9 months, respectively. There was a decrease in PR3-ANCA at 2 months in 93 (71%), 50 (38%) and 120 (92%) of patients measured by direct ELISA, capture ELISA, and ALBIA, respectively. Of the 131 participants, 108 met criteria for complete remission at some point during follow-up and 92 met criteria for sustained remission. Those patients who had a decrease in PR3-ANCA by ALBIA at 2 months had a shorter time to sustained remission (HR 4.52, 95% CI 1.11, 18.42, *p* = 0.035) (Table 1). In patients who had a decrease in PR3-ANCA by ALBIA at 2 months, the median time to sustained remission was 12 months (9 and 12 months for RTX and CYC, respectively). Among the 11 patients (4 RTX, 7 CYC) who did not have a decrease in PR3-ANCA by ALBIA at 2 months, only 2 patients (both RTX) achieved sustained remission during follow-up.

**Table 1:** Association between a decrease in PR3-ANCA and time to remission.

	**Complete remission^‡^**			**Sustained remission^‡^**		
	**HR**	**95% C.I**.	***p***	**HR**	**95% C.I**.	***p***
Overall (N^*^= 131)						
**Decrease by 2 months**						
Direct (*n*^†^ = 93)	1.51	(0.95, 2.40)	0.079	1.45	(0.90, 2.42)	0.139
Capture(*n*^†^ = 50)	1.26	(0.86, 1.84)	0.245	1.14	(0.75, 1.72)	0.541
ALBIA(*n*^†^ = 120)	2.11	(0.86, 5.22)	0.104	4.52	(1.11, 18.42)	0.035
**Decrease by 4 months**						
Direct (*n*^†^ =101)	1.75	(1.03, 2.97)	0.038	1.77	(1.03, 3.28)	0.050
Capture (*n*^†^ = 68)	1.39	(0.94, 2.05)	0.102	1.29	(0.86, 1.98)	0.225
ALBIA(*n*^†^ = 121)	1.73	(0.70, 4.26)	0.234	3.81	(0.94, 15.53)	0.062
**Negative by 4 months**						
Direct (*n*^†^ = 74)	1.07	(0.73, 1.58)	0.729	1.00	(0.66, 1.51)	0.996
Capture (*n*^†^ = 41)	1.18	(0.80, 1.75)	0.413	0.97	(0.62, 1.50)	0.881
ALBIA (*n*^†^ = 55)	1.08	(0.73, 1.57)	0.721	0.98	(0.65, 1.49)	0.939

*Total number of patients.

† *Number of patients who experienced the given decrease in ANCA by the stated time period*.

‡ *Separate analyses were performed for each remission definition and ANCA decrease definition using proportional hazards regression. Findings are summarized using the hazard ratio (HR) and corresponding 95% confidence interval. A hazard ratio significantly >1.0 indicates that experiencing the given ANCA decrease by the given time period is associated with a shorter time to achieving the given remission endpoint*.

A decrease in PR3-ANCA at 4 months occurred in 101 (77%), 68 (52%), and 121 (92%) of patients by direct ELISA, capture ELISA, and ALBIA, respectively. A decrease in PR3-ANCA by direct ELISA at 4 months was associated with decreased time to sustained remission (HR 1.77, 95% CI 1.03, 3.28) (Table 1) with a median time to sustained remission of 9 months in those patients who had a decrease in PR3-ANCA by direct ELISA at 4 months compared to 12 months in those who did not.

PR3-ANCA negativity occurred at 4 months in 74 (56%), 41 (31%), and 55 (42%) patients by direct ELISA, capture ELISA and ALBIA, respectively. There was no association between PR3-ANCA negativity at 4 months by any PR3-ANCA assay and time to complete or sustained remission (Table 1).

When stratified by treatment group there was an association between a decrease in PR3-ANCA by ALBIA at 2 and 4 months and time to sustained remission in patients treated with CYC/AZA (Table 2).

**Table 2:** Association between a decrease in PR3-ANCA and time to remission—by Treatment.

	**Complete remission^‡^**			**Sustained remission^‡^**		
	**HR**	**95% C.I**.	***p***	**HR**	**95% C.I**.	***p***
**RTX (**N^*^= **66)**						
**Decrease by 2 months**						
Direct (*n*^†^ = 50)	1.41	(0.67, 2.97)	0.360	1.41	(0.66, 3.02)	0.381
Capture (*n*^†^ = 26)	1.17	(0.68, 1.99)	0.575	1.06	(0.59, 1.89)	0.853
ALBIA (*n*^†^ = 62)	2.05	(0.49, 8.53)	0.322	1.28	(0.31, 5.27)	0.735
**Decrease by 4 months**						
Direct (*n*^†^ = 55)	1.62	(0.62, 4.21)	0.323	1.85	(0.66, 5.17)	0.241
Capture (*n*^†^ = 38)	1.25	(0.71, 2.21)	0.442	1.19	(0.65, 2.19)	0.567
ALBIA (*n*^†^ = 63)	1.16	(0.28, 4.77)	0.841	0.71	(0.17, 2.95)	0.642
**Negative by 4 months**						
Direct (*n*^†^ = 39)	1.10	(0.62, 1.92)	0.752	0.97	(0.53, 1.76)	0.910
Capture (*n*^†^ = 19)	1.19	(0.67, 2.08)	0.555	1.04	(0.55, 1.94)	0.912
ALBIA (*n*^†^ = 29)	1.04	(0.61, 1.76)	0.893	0.96	(0.54, 1.71)	0.883
**CYC (**N^*^= **65)**
**Decrease by 2 months**						
Direct (*n*^†^ = 43)	1.42	(0.78, 2.60)	0.255	1.46	(0.77, 2.78)	0.250
Capture (*n*^†^ = 24)	1.29	(0.74, 2.23)	0.373	1.24	(0.69, 2.23)	0.475
ALBIA (*n*^†^ = 58)	2.06	(0.64, 6.63)	0.228	∞	(3.84, ∞)	<0.001
**Decrease by 4 months**						
Direct (*n*^†^ = 46)	1.63	(0.85, 3.13)	0.140	1.75	(0.86, 3.54)	0.121
Capture (*n*^†^ = 30)	1.37	(0.79, 2.36)	0.265	1.38	(0.77, 2.46)	0.282
ALBIA (*n*^†^ = 58)	2.06	(0.64, 6.63)	0.228	∞	(3.84, ∞)	<0.001
**Negative by 4 months**						
Direct (*n*^†^ = 35)	0.96	(0.56, 1.66)	0.894	1.02	(0.57, 1.81)	0.960
Capture (*n*^†^ = 22)	1.16	(0.66, 2.03)	0.599	0.89	(0.48, 1.65)	0.710
ALBIA (*n*^†^ = 26)	1.04	(0.60, 1.82)	0.880	0.98	(0.54, 1.79)	0.948

* *Total number of patients in the given treatment group*.

† *Number of patients in the given treatment group who experienced the given decrease in ANCA by the stated time period*.

‡ *Separate analyses were performed for each remission definition and ANCA decrease definition using proportional hazards regression. Findings are summarized using the hazard ratio (HR) and corresponding 95% confidence interval. A hazard ratio significantly >1.0 indicates that experiencing the given ANCA decrease by the given time period is associated with a shorter time to achieving the given remission endpoint*.

A decrease in PR3-ANCA by direct or capture ELISA at 4 months was associated with decreased time to complete remission in patients with granulomatous disease (Table 3). There was no other association between PR3-ANCA decrease or negativity and time to complete or sustained remission when stratified by treatment group or disease phenotype (Tables 2, 3).

**Table 3:** Association between a decrease in PR3 ANCA and time to remission—by Phenotype.

	**Complete remission^‡^**			**Sustained remission^‡^**		
	**HR**	**95% C.I**.	***p***	**HR**	**95% C.I**.	***p***
**Renal Involvement (**N^*^= **78)**						
**Decrease by 2 months**
Direct (*n*^†^ =56)	1.08	(0.61, 1.91)	0.801	1.20	(0.65, 2.19)	0.563
Capture (*n*^†^ = 30)	1.09	(0.67, 1.77)	0.735	0.94	(0.55, 1.59)	0.809
ALBIA (*n*^†^ = 72)	1.80	(0.56, 5.78)	0.323	2.08	(0.51, 8.56)	0.310
**Decrease by 4 months**						
Direct (*n*^†^ = 62)	1.40	(0.70, 2.79)	0.346	1.65	(0.78, 3.50)	0.191
Capture (*n*^†^ = 39)	1.27	(0.78, 2.08)	0.341	1.00	(0.60, 1.69)	0.992
ALBIA (*n*^†^ = 73)	1.12	(0.35, 3.59)	0.848	1.39	(0.34, 5.70)	0.647
**Negative by 4 months**						
Direct (*n*^†^ = 41)	1.03	(0.63, 1.69)	0.900	1.05	(0.62, 1.77)	0.849
Capture (*n*^†^ = 24)	1.05	(0.63, 1.75)	0.846	0.83	(0.47, 1.46)	0.513
ALBIA (*n*^†^ = 29)	1.11	(0.68, 1.83)	0.678	1.04	(0.61, 1.79)	0.878
**Capillaritis (**N^*^= **105)**						
**Decrease by 2 months**						
Direct (*n*^†^ = 76)	1.13	(0.69, 1.85)	0.631	1.15	(0.67, 1.95)	0.617
Capture (*n*^†^ = 41)	1.06	(0.69, 1.61)	0.792	0.98	(0.62, 1.55)	0.922
ALBIA (*n*^†^ = 95)	1.90	(0.76, 4.71)	0.169	3.96	(0.97, 16.23)	0.056
**Decrease by 4 months**						
Direct (*n*^†^ = 83)	1.39	(0.78, 2.48)	0.270	1.51	(0.80, 2.87)	0.208
Capture (*n*^†^ = 54)	1.24	(0.81, 1.90)	0.330	1.13	(0.71, 1.79)	0.604
ALBIA (*n*^†^ = 96)	1.49	(0.60, 3.69)	0.391	3.24	(0.79, 13.26)	0.102
**Negative by 4 months**						
Direct (*n*^†^ = 59)	1.01	(0.66, 1.54)	0.979	0.97	(0.61, 1.54)	0.896
Capture (*n*^†^ = 34)	1.03	(0.66, 1.58)	0.911	0.84	(0.51, 1.37)	0.478
ALBIA (n = 42)	1.04	(0.68, 1.59)	0.862	0.99	(0.62, 1.58)	0.975
**DAH (**N^*^= **38)**						
**Decrease by 2 months**						
Direct (*n*^†^ = 30)	1.65	(0.62, 4.35)	0.313	1.34	(0.46, 3.92)	0.596
Capture (*n*^†^ = 12)	0.81	(0.38, 1.73)	0.582	0.86	(0.37, 2.01)	0.727
ALBIA (*n*^†^ = 37)	1.46	(0.19, 10.98)	0.713	∞	(0.49, ∞)	0.164
**Decrease by 4 months**						
Direct (*n*^†^ = 33)	3.02	(0.70, 13.05)	0.139	3.85	(0.52, 28.69)	0.189
Capture (*n*^†^ = 18)	0.95	(0.46, 1.94)	0.885	0.97	(0.43, 2.15)	0.935
ALBIA (*n*^†^ = 37)	1.46	(0.19, 10.98)	0.713	∞	(0.49, ∞)	0.164
**Negative by 4 months**						
Direct (*n*^†^ = 21)	1.33	(0.63, 2.80)	0.459	0.91	(0.40, 2.05)	0.820
Capture (*n*^†^ = 11)	0.80	(0.37, 1.75)	0.574	0.48	(0.18, 1.29)	0.145
ALBIA (*n*^†^ = 17)	1.27	(0.62, 2.61)	0.518	1.12	(0.50, 2.51)	0.778
**Granulomatous (**N^*^= **102)**						
**Decrease by 2 months**						
Direct (*n*^†^ = 75)	1.57	(0.91, 2.70)	0.108	1.44	(0.81, 2.55)	0.212
Capture (*n*^†^ = 42)	1.41	(0.91, 2.17)	0.124	1.35	(0.84, 2.15)	0.213
ALBIA (*n*^†^ = 94)	3.10	(0.97, 9.95)	0.057	3.22	(0.79, 13.15)	0.104
**Decrease by 4 months**						
Direct (*n*^†^ = 79)	1.95	(1.06, 3.58)	0.033	1.80	(0.94, 3.43)	0.076
Capture (*n*^†^ = 55)	1.68	(1.07, 2.64)	0.026	1.57	(0.97, 2.54)	0.068
ALBIA (*n*^†^ = 95)	2.41	(0.75, 7.71)	0.138	2.53	(0.62, 10.32)	0.197
**Negative by 4 months**						
Direct (*n*^†^ = 63)	0.97	(0.62, 1.50)	0.874	0.86	(0.53, 1.37)	0.518
Capture (*n*^†^ = 34)	1.29	(0.83, 2.00)	0.260	1.10	(0.68, 1.80)	0.694
ALBIA (*n*^†^ = 47)	1.02	(0.66, 1.56)	0.936	0.89	(0.56, 1.42)	0.629

* *Total number of patients with the given phenotype*.

† *Number of patients with the given phenotype who experienced the given decrease in ANCA by the stated time period*.

‡ *Separate analyses were performed for each remission definition and ANCA decrease definition using proportional hazards regression. Findings are summarized using the hazard ratio (HR) and corresponding 95% confidence interval. A hazard ratio significantly >1.0 indicates that experiencing the given ANCA decrease by the given time period is associated with a shorter time to achieving the given remission endpoint*.

### PR3-ANCA Titer Increases Determined by ALBIA and Subsequent Relapse

The baseline characteristics of the 93 PR3-ANCA positive patients who achieved complete remission have been reported elsewhere (35). Relapses occurred in 55 of the 93 subjects (59%). An increase in PR3-ANCA by ALBIA was found in 78 of 93 subjects (84%). Of these patients 11 (14%) had a PR3-ANCA value >4 units which subsequently increased to a value >8 units and therefore required titration for the detection of the increase. Four of these 11 patients experienced a subsequent relapse. Relapse occurred concurrently or after a rise in PR3-ANCA by ALBIA in 47 of the 55 relapses (85%) and within 1 year of an increase in 29 of the 55 relapses (53%). The median time to any relapse after PR3-ANCA increase was 15.4 months. Kaplan-Meier estimates for time to relapse following an increase in PR3-ANCA level stratified by disease phenotype and treatment arms are shown in Table 4. The number of patients with rise in PR3-ANCA followed by a relapse and time to relapse for the entire cohort with categorization by severity of relapse, disease phenotype, and treatment received are shown in the Supplementary Figure.

**Table 4:** Kaplan-Meier estimates for relapse following a rise in PR3-ANCA^*^.

	**ALBIA**				
		**Median months to relapse**	**Cumulative Relapse, % (95% C.I.)**		
	N^*^		**6-months**	**12 months**	**18 months**
**Overall**					
Any relapse	72	15.4	26 (15, 35)	42 (29, 52)	52 (38, 62)
Severe relapse	80	22.1	22 (12, 30)	35 (23, 45)	42 (30, 52)
**Baseline capillaritis**					
Any relapse	58	22.1	23 (11, 33)	36 (22, 48)	44 (29, 56)
Severe relapse	66	24.2	19 (8, 28)	32 (19, 42)	37 (24, 48)
**Baseline renal**					
Any relapse	44	–	26 (11, 38)	31 (15, 44)	39 (22, 53)
Severe relapse	51	37.0	20 (8, 31)	29 (15, 41)	36 (20, 52)
**Baseline DAH**					
Any relapse	20	10.2	43 (15, 61)	54 (24, 72)	67 (35, 83)
Severe relapse	22	14.3	34 (10, 51)	49 (22, 67)	60 (31, 77)
**Rituximab group**					
Any relapse	37	14.6	22 (7, 35)	42 (24, 57)	55 (34, 69)
Severe relapse	44	21.7	21 (8, 32)	35 (19, 48)	45 (28, 59)
**Cyclophosphamide**					
Any relapse	35	18.2	29 (12, 42)	41 (22, 56)	48 (28, 63)
Severe relapse	36	24.2	22 (7, 35)	34 (16, 48)	38 (19, 52)

* *Analyses include individuals who experienced a rise in PR3-ANCA during follow-up while at risk for the given relapse endpoint. Individuals who experienced an ANCA increase concurrent with the given relapse event are not included. Time zero corresponds to the date of the ANCA increase*.

Of the 15 patients who did not experience an increase in PR3-ANCA, 8 (53%) developed a subsequent relapse (Supplementary Figure). Additionally, of the 78 patients who had an increase in PR3-ANCA, 31 (40%) did not experience a relapse. Five of these 31 had <1 year of follow-up after PR3-ANCA increase. Twenty patients had a PR3-ANCA increase but then had a subsequent decrease. Of these patients who had an initial increase but then a decrease, 3 experienced a relapse.

An increase of PR3-ANCA levels determined by ALBIA was associated with subsequent severe relapse (*p* = 0.002). This association was true for the subgroups of patients with renal involvement, capillaritis, diffuse alveolar hemorrhage, and those treated with RTX (Table 5). These results are comparable to the previously reported data on the utility of direct ELISA to predict relapse. Of the 42 severe relapses that occurred in this patient cohort, 39 (93%) had a preceding increase in PR3-ANCA (Supplementary Figure).

**Table 5:** Proportional hazards regression assessing whether an increase in ANCA is associated with relapse (Truncated after 1-year).

	**HR**	**95% C.I**.	***p***	**c-index**
**All subjects**				
Any flare	1.57	0.81, 3.05	0.156	0.55
Severe flare	5.45	1.83, 16.19	0.002	0.65
**According to treatment**				
**Cyclophosphamide**				
Any flare	1.64	0.50, 5.41	0.418	0.52
Severe flare	8.04	0.97, 66.47	0.053	0.62
**Rituximab**				
Any flare	1.59	0.65, 3.86	0.307	0.54
Severe flare	5.58	1.11, 28.04	0.037	0.64
**According to renal involvement**				
**Without renal involvement**				
Any flare	2.67	0.83, 8.56	0.100	0.60
Severe flare	3.04	0.80, 11.54	0.103	0.63
**With renal involvement**				
Any flare	1.09	0.47, 2.54	0.844	0.50
Severe flare	10.32	1.32, 80.97	0.026	0.67
**According to baseline capillaritis**				
**Without capillaritis**				
Any flare	4.74	0.53, 42.52	0.165	0.63
Severe flare	4.38	0.47, 40.42	0.193	0.64
**With capillaritis**				
Any flare	1.23	0.60, 2.54	0.573	0.52
Severe flare	5.43	1.55, 19.11	0.008	0.66
**According to baseline DAH**				
**Without DAH**				
Any flare	0.96	0.45, 2.06	0.918	0.50
Severe flare	2.87	0.91, 9.06	0.072	0.61
**With DAH**				
Any flare	5.24	1.13, 24.26	0.034	0.68
Severe flare	∞	4.21, ∞	<0.001	0.74
**According to new vs. relapsing**				
**Relapsing disease**				
Any flare	1.24	0.54, 2.83	0.613	0.51
Severe flare	6.62	1.43, 30.79	0.016	0.65
**New disease**				
Any flare	2.51	0.68, 9.27	0.166	0.59
Severe flare	7.18	0.89, 58.03	0.065	0.66
**Any granulomatous disease**				
Any flare	1.89	0.81, 4.24	0.143	0.56
Severe flare	7.22	1.64, 31.75	0.009	0.64
**Granulomatous disease only**				
Any flare	2.88	0.28, 30.00	0.375	0.59
Severe flare	2.88	0.28, 30.00	0.375	0.61

The effect of disease phenotype on PR3-ANCA increase and relapse association was also investigated. For the association of PR3-ANCA increase to severe relapse, the c-indices ranged from 0.61 in patients with granulomatous disease only to 0.74 in patients with DAH, the subgroup in which the most significant association was observed (Table 5). The median time to any relapse following an increase in PR3-ANCA in patients with DAH was 10.2 months (Table 4). In this subgroup, there were no relapse that occurred without a preceding PR3-ANCA increase (Supplementary Figure).

There was also a difference amongst treatment groups in the association between PR3-ANCA increase and relapse. This association was stronger among patients treated with RTX compared to those treated with CYC/AZA (Table 5). In the patients who were treated with RTX, 46% (23 of 50) experienced a severe relapse. Ninety-six percent (22 of 23) of these relapses were preceded by a rise in PR3-ANCA in this group. In comparison, 44% (19 of 43) patients treated with CYC/AZA experienced a severe relapse and 89% (17 of 19) were preceded by an increase in PR3-ANCA (Supplementary Figure).

## Discussion

The diagnostic utility of ANCA testing for vasculitis is well-established, and the revised 2017 international consensus statement of ANCA testing in GPA and MPA has summarized results obtained with various state-of-the-art antigen-specific ANCA test methodologies including ALBIA, and recommends their use as primary diagnostic ANCA tests for GPA and MPA (48). Automated ALBIA platforms use glass, latex or magnetic beads to immobilize the antigen or antibody of interest. Light scatter and fluorescence are then used to obtain antibody measurements (45). Vasculitis specific commercial automated ALBIAs can measure multiple antibodies of interest from the same serum sample in a single tube including PR3-ANCA, MPO-ANCA, and anti-glomerular basement membrane (anti-GBM) antibody. The agreement between automated ALBIAs with immunofluorescence and commercially available ELISA kits have been shown to be high (45). Given the relative ease of this assay without compromising analytic sensitivity or specificity, many high volume clinical laboratories now utilize automated ALBIAs for detection of ANCA.

The 2017 consensus statement does not address the clinical utility of serial ANCA testing as a biological marker of AAV disease activity (48). This has been controversial for years (11–34). Using mostly indirect immunofluorescence to determine ANCA titers or ELISA methods, several studies have suggested that a decrease in ANCA titer during induction therapy may be indicative of disease response (15, 20, 26) while others using capture ELISA did not find a clear association (27). The present study demonstrated a decrease in PR3-ANCA by ALBIA at 2 months was associated with decreased time to sustained remission. This association was strongest in patients treated with CYC/AZA. In patients treated with CYC/AZA who did not have a decrease in PR3-ANCA by ALBIA, none achieved sustained remission. The reason no significant association was observed for patients treated with RTX may be explained by the fact that most of these patients had a decrease (62 and 63 of the 66 patients by 2 and 4 months, respectively). Hence, a lack of PR3-ANCA decrease by ALBIA at 2 months may be predictive of refractory disease. Clinicians should closely monitor these patients and be prepared to change therapy if the clinical response is delayed or incomplete.

A prior study demonstrated a possible association of PR3-ANCA increases determined by direct ELISA with subsequent relapse during serial follow-up of patients (35). This association was not found when the capture ELISA was used for PR3-ANCA detection (35). The present study examined the utility of an automated ALBIA in predicting relapse in AAV patients in comparison to methods previously reported. The present study replicates the previous findings obtained by direct ELISA that PR3-ANCA may have clinical utility in relapse prediction with limitations (35). It was found that an increase in PR3-ANCA has the strongest association with relapses of AAV in patients who: experience a severe relapse, have severe disease manifestations caused by capillaritis such as diffuse alveolar hemorrhage or renal involvement, and in patients treated with rituximab. In the present study, there was no association between a PR3-ANCA increase and relapse in patients with isolated necrotizing granulomatous inflammation at baseline, further supporting the stronger correlation between PR3-ANCA and capillaritis compared to PR3-ANCA and granulomatous inflammation seen in past studies (34, 35). *In vitro* and *in vivo* studies also provide support for this observation (49–52). ANCA has been shown to induce neutrophil activation that leads to capillaritis manifestations *in vitro* (49, 50). In some proposed animal models of PR3-ANCA disease, capillaritis also develops but convincing evidence of granulomatous inflammation has not been reported to date (51–53). The association of PR3-ANCA increase and subsequent relapse was consistent in all patients with capillaritis at disease presentation, regardless of treatment regimen received.

There was a stronger association of PR3-ANCA increase and relapse in patients treated with RTX compared to those treated with CYC/AZA. This may have several reasons. First, PR3-ANCA positive patients are more likely to turn ANCA negative when treated with RTX than when treated with CYC for remission induction (3). Second, per study protocol, patients in the RTX arm did not receive additional maintenance agents such as AZA or methotrexate after induction with RTX with four weekly treatments of 375 mg/m^2^ (3). Thus, B cell depleting therapy seems to be suppressing ANCA production more effectively than therapy that merely suppresses the B cell numbers. Conversely, when ANCA production resumes as B cells reconstitute a PR3-ANCA level increase may be more vigorous and more clearly identify patients at risk for relapse.

It is important to note that not all patients who had a rise in PR3-ANCA had a subsequent relapse. This was true even amongst patients with phenotypes and treatment regimens where PR3-ANCA and relapse were strongly associated with subsequent relapse such as those with capillaritis and those treated with RTX induction therapy. The risk of relapse therefore needs to be weighed against the side effects of treatment with individual patient factors considered. It is also interesting to note that among 12 patients who did not have an increase in PR3-ANCA, 9 (75%) did not experience a severe relapse during follow-up.

This study reconfirms that different ANCA detection assays perform differently when applied in clinical practice. This is why it is important for clinicians to know how each assay performs. The differences between assays are most likely the result of the PR3 antigen being presented differently in the solid phase in the different assays. Since ANCA are polyclonal antibodies it is not surprising that these antibodies recognizing different epitopes bind differently in the various assays which present the antigen in different fashions. This is why it is important to know what assays can be used clinically. The current findings expand the acceptable PR3-ANCA testing methods for disease monitoring to include the more convenient, routinely used automated ALBIA technology. It is important to note that without titration of the ALBIA values, 14% of the increases detected would have been missed. It is therefore strongly suggested that the serum samples be titrated when the automated ALBIA is used for AAV disease monitoring. In this study values considered equivocal in the ALBIA assay (0.4–0.9) had the cANCA pattern confirmed by immunofluorescence. Confirmation by immunofluorescence or another ANCA assay should be completed for equivocal values if using the ALBIA assay as a high-volume screening assay to increase the sensitivity without compromising specificity.

There are several limitations to this study. Classifications of disease phenotype and severity of relapse were based on BVAS/WG forms completed during the RAVE trial by expert clinicians. This data was unable to be verified at the time of this study. Disease phenotype was classified based on disease activity at baseline and not adjusted for changes in phenotype at relapse. The intervals of ANCA measurement were in accordance with the RAVE study protocol (3). For the time period following the first 6 months of remission induction therapy, this consisted of ANCA measurement every 3 months until month 18 from enrollment, followed by ANCA measurement every 6 months until completion of the study. With these set intervals it is possible that changes in ANCA titer may have been missed as it has been suggested that more frequent ANCA testing is better associated with disease activity prediction (30). Only PR3-ANCA positive patients were analyzed for this study. This was due to the established increased risk of relapse in this population compared to MPO-ANCA positive patients (7, 33, 36–41). A much higher number of MPO-ANCA positive patients would have to be followed in order to study the relationship between ANCA levels and disease relapse in MPO-ANCA positive patients. The current study involved a large set of analyses of the association of changes in ANCA levels determined by different detection methods with various subsequent outcomes, in two treatment groups. Thus, some of the statistically significant findings, both positive and negative, may be due to chance. Prior to incorporating any of these findings into clinical management strategies, the results of this study should be confirmed in an independent cohort using similar techniques.

## Conclusions

A PR3-ANCA decrease by ALBIA at 2 months was associated with decreased time to sustained remission and may be predictive of refractory disease. Measurement of PR3-ANCA by ALBIA at 2 months may help clinicians identify those patients who will not respond to the current therapy. Clinicians should consider changing to an alternative therapy or close monitoring of patients without a decrease in PR3-ANCA by ALBIA at 2 months. ALBIA for PR3-ANCA measurement is comparable to direct ELISA in predicting relapse in PR3-AAV. Therefore, ALBIA which is a widely used ANCA assay in many high-volume laboratories can be used for serial ANCA testing for disease monitoring with no need to change to direct ELISA for disease monitoring. It is important to note without titration, 14% of the increases detected by ALBIA would have been missed. Consequently, titration is recommended when this assay is used for disease monitoring in AAV. Our study has limitations, and the results should be confirmed in a separate cohort. The association of an increase in PR3-ANCA with the risk of subsequent relapse remains complex and is affected by disease phenotype and remission induction agent. Individual patient factors need to be considered when applying this information clinically.

## Data Availability Statement

The raw data supporting the conclusions of this article will be made available by the authors, without undue reservation.

## Ethics Statement

The studies involving human participants were reviewed and approved by Institutional Review Board, Mayo Clinic, Rochester, MN. The patients/participants provided their written informed consent to participate in this study.

## Author Contributions

GT completed data organization and analysis, writing of manuscript. AH, FS, and MS generated the ANCA data. DS completed statistical analysis, generation of tables. LF, CL, PMe, PMo, PS, RS, ES, JS, and US were involved in the RAVE trial design and completed the clinical data collection. US designed the present project, completed data collection, writing of manuscript. All authors were involved with editing the manuscript and approved the final version.

## Conflict of Interest

The authors declare that this study received funding from Genentech, Inc. and Biogen IDEC, Inc. The funders were not involved in the study design, collection, analysis, interpretation of data, the writing of this article or the decision to submit it for publication.
