# Supplementary material for: Clinical Utility of Serial Measurements of Antineutrophil Cytoplasmic Antibodies Targeting Proteinase 3 in ANCA-Associated Vasculitis
Source: Front Immunol. 2020 Sep 3;11:2053. doi: 10.3389/fimmu.2020.02053 (PMC7495134; doi:10.3389/fimmu.2020.02053)
Supplement: Supplementary file 1 [file Table_1.DOCX]

Supplementary Figure: PR3-ANCA followed by a relapse and time to relapse with categorization by severity of relapse, disease phenotype, and treatment received

Any Relapse according to PR3-ANCA level increase - Overall

| Bead-based assay (N=93) | | |
| --- | --- | --- |
| PR3-ANCA increase  (N=78) |  | No PR3-ANCA increase (N=15) |
| Relapse (N=47) |  | Relapse (N=8) |
| Concurrent (N=6) |  |  |
| ≤ 1 year (N=29) |  |  |
| > 1 Year (N=12) |  |  |
| No Relapse (N=31)† |  | No Relapse (N=7)†† |
| † 5 of the 31 had < 1 year of follow-up after PR3-ANCA increase  †† 1 of the 7 had < 1 year of follow-up after achieving remission | | |

Severe Relapse according to PR3-ANCA level increase - Overall

| Bead-based assay (N=93) | | |
| --- | --- | --- |
| PR3-ANCA increase  (N=81) |  | No PR3-ANCA increase (N=12) |
| Relapse (N=39) |  | Relapse (N=3) |
| Concurrent (N=1) |  |  |
| ≤ 1 year (N=26) |  |  |
| > 1 Year (N=12) |  |  |
| No Relapse (N=42)† |  | No Relapse (N=9)†† |
| † 6 of the 42 had < 1 year of follow-up after PR3-ANCA increase  †† 1 of the 9 had < 1 year of follow-up after achieving remission | | |

Any Relapse according to PR3-ANCA level increase in those treated with CYC (N=43)

| Bead-based assay | | |
| --- | --- | --- |
| PR3-ANCA increase  (N=35) |  | No PR3-ANCA increase  (N=8) |
| Relapse (N=19) |  | Relapse (N=5) |
| Concurrent (N=0) |  |  |
| ≤ 1 year (N= 14) |  |  |
| > 1 Year (N= 5) |  |  |
| No Relapse (N=16)† |  | No Relapse (N=3)†† |
| † 3 of the 16 had < 1 year of follow-up after PR3-ANCA increase  †† 0 of the 3 had < 1 year of follow-up after achieving remission | | |

Severe Relapse according to PR3-ANCA level increase in those treated with CYC (N=43)

| Bead-based assay | | |
| --- | --- | --- |
| PR3-ANCA increase  (N=36) |  | No PR3-ANCA increase  (N=7) |
| Relapse (N=17) |  | Relapse (N=2) |
| Concurrent (N=0) |  |  |
| ≤ 1 year (N= 12) |  |  |
| > 1 Year (N= 5) |  |  |
| No Relapse (N=19)† |  | No Relapse (N=5)†† |
| † 3 of the 19 had < 1 year of follow-up after PR3-ANCA increase  †† 0 of the 5 had < 1 year of follow-up after achieving remission | | |

Any Relapse according to PR3-ANCA level increase in those treated with RTX (N=50)

| Bead-based assay | | |
| --- | --- | --- |
| PR3-ANCA increase  (N=43) |  | No PR3-ANCA increase  (N=7) |
| Relapse (N=28) |  | Relapse (N=3) |
| Concurrent (N=6) |  |  |
| ≤ 1 year (N=15) |  |  |
| > 1 Year (N=7) |  |  |
| No Relapse (N=15)† |  | No Relapse (N=4)†† |
| † 2 of the 15 had < 1 year of follow-up after PR3-ANCA increase  †† 1 of the 4 had < 1 year of follow-up after achieving remission | | |

Severe Relapse according to PR3-ANCA level increase in those treated with RTX (N=50)

| Bead-based assay | | |
| --- | --- | --- |
| PR3-ANCA increase  (N=45) |  | No PR3-ANCA increase  (N=5) |
| Relapse (N=22) |  | Relapse (N=1) |
| Concurrent (N=1) |  |  |
| ≤ 1 year (N=14) |  |  |
| > 1 Year (N=7) |  |  |
| No Relapse (N=23)† |  | No Relapse (N=4)†† |
| † 3 of the 23 had < 1 year of follow-up after PR3-ANCA increase  †† 1 of the 4 had < 1 year of follow-up after achieving remission | | |

Any Relapse according to PR3-ANCA level increase in those with baseline DAH N=24 (8 CYC, 16 RTX)

| Bead-based assay | | |
| --- | --- | --- |
| PR3-ANCA increase  N=22 (7, 15) |  | No PR3-ANCA increase  N=2 (1, 1) |
| Relapse N=14 (4, 10) |  | Relapse N=0 (0, 0) |
| Concurrent N=2 (0, 2) |  |  |
| ≤ 1 year N=10 (4, 6) |  |  |
| > 1 Year N=2 (0, 2) |  |  |
| No Relapse N=8 (3, 5) † |  | No Relapse N=2 (1, 1) †† |
| † 2 (1, 1) of the 8 (3, 5) had < 1 year of follow-up after PR3-ANCA increase  †† 0 (0, 0) of the 2 (1, 1) had < 1 year of follow-up after achieving remission | | |

Severe Relapse according to PR3-ANCA level increase in those with baseline DAH N=24 (8 CYC, 16 RTX)

| Bead-based assay | | | |
| --- | --- | --- | --- |
| PR3-ANCA increase  N=22 (7, 15) |  | No PR3-ANCA increase  N=2 (1, 1) | |
| Relapse N=11 (4, 7) |  | Relapse N=0 (0, 0) | |
| Concurrent N=0 (0, 0) |  |  | |
| ≤ 1 year N=10 (4, 6) |  |  | |
| > 1 Year N=1 (0, 1) |  |  | |
| No Relapse N=11 (3, 8) † |  | No Relapse N=2 (1, 1) †† | |
| † 2 (1, 1) of the 11 (3, 8) had < 1 year of follow-up after PR3-ANCA increase  †† 0 (0, 0) of the 2 (1, 1) had < 1 year of follow-up after achieving remission | | |  |

Any Relapse according to PR3-ANCA level increase in those with baseline Renal involvement N=60 (29 CYC, 31 RTX)

| Bead-based assay | | | |
| --- | --- | --- | --- |
| PR3-ANCA increase  N=49 (23, 26) |  | No PR3-ANCA increase  N=11 (6, 5) | |
| Relapse N=23 (8, 15) |  | Relapse N=5 (3, 2) | |
| Concurrent N=5 (0, 5) |  |  | |
| ≤ 1 year N=13 (7, 6) |  |  | |
| > 1 Year N=5 (1, 4) |  |  | |
| No Relapse N=26 (15, 11) † |  | No Relapse N=6 (3, 3) †† | |
| † 5 (3, 2) of the 26 (15, 11) had < 1 year of follow-up after PR3-ANCA increase  †† 1 (0, 1) of the 6 (3, 3) had < 1 year of follow-up after achieving remission | | |  |

Any Relapse according to PR3-ANCA level increase in those with baseline capillaritis N=77 (37 CYC, 40 RTX)

| Bead-based assay | | |
| --- | --- | --- |
| PR3-ANCA increase  N=63 (29, 34) |  | No PR3-ANCA increase  N=14 (8, 6) |
| Relapse N=34 (13, 21) |  | Relapse N=8 (5, 3) |
| Concurrent N=5 (0, 5) |  |  |
| ≤ 1 year N=20 (10, 10) |  |  |
| > 1 Year N=9 (3, 6) |  |  |
| No Relapse N=29 (16, 13) † |  | No Relapse N=6 (3, 3) †† |
| † 5 (3, 2) of the 29 (16, 13) had < 1 year of follow-up after PR3-ANCA increase  †† 1 (0, 1) of the 6 (3, 3) had < 1 year of follow-up after achieving remission | | |

Any Relapse according to PR3-ANCA level increase in those with baseline granulomatous only N=15 (6 CYC, 9 RTX)

| Bead-based assay | | |
| --- | --- | --- |
| PR3-ANCA increase  N=15 (6, 9) |  | No PR3-ANCA increase  N=0 (0, 0) |
| Relapse N=13 (6, 7) |  |  |
| Concurrent N=1 (0, 1) |  |  |
| ≤ 1 year N=9 (4, 5) |  |  |
| > 1 Year N=3 (2, 1) |  |  |
| No Relapse N=2 (0, 2) † |  |  |
| † Neither patient had < 1 year of follow-up after PR3-ANCA increase | | |
